# Brucellosis in food-producing animals in Mosul, Iraq: A systematic review and meta-analysis

**DOI:** 10.1371/journal.pone.0235862

**Published:** 2020-07-09

**Authors:** Mohammad O. Dahl

**Affiliations:** Department of Internal and Preventive Medicine, College of Veterinary Medicine, University of Mosul, Mosul, Iraq; East Carolina University Brody School of Medicine, UNITED STATES

## Abstract

Brucellosis is an endemic disease in food-producing animals in Mosul, Iraq. The objectives of the study reported here were: (i) to identify and assess the evidence and knowledge gaps in published studies that have examined brucellosis in different food-producing animals in Mosul, Iraq; using systematic review approach, and (ii) to quantify the seroprevalence of brucellosis in the city using meta-analysis approach. Google Scholar was used as a search engine to track pertinent peer-reviewed research reports. The search was conducted on November 24, 2019. Keywords used were: brucella, animal, Mosul, Iraq. Peer-reviewed published studies, MSc theses, and PhD dissertations written in Arabic or English were included. Duplicate records were removed, and the screening process was conducted at three levels: titles, abstracts, and full-text articles. Identified studies that have reported the seroprevalence of brucellosis were included in a meta-analysis to calculate an overall prevalence. A total of 214 records were initially identified. Seventeen research reports were added from personal contact and qualified articles’ references list. Thirty six articles were qualified for review after removing 35 duplicate records, 155 titles, 11 abstracts, and 5 full text articles. Seventeen studies reported the prevalence of brucellosis, 11 studies assessed different serological tests for diagnosis of brucellosis, 9 studies isolated *Brucella* spp. from animal specimens and/or animal products, and 4 studies assessed vaccination procedures against brucellosis. The overall seroprevalence of brucellosis in food-producing animals in Mosul over a period of 40 years was 14.14%, including 14.46% for sheep, 12.99% for goats, 11.69% for cattle, and 22.64% for buffalo. The study concluded that the disease is evident in the city with increasing trends over the years, buffalo shows high seroprevalence, the degree of agreement of Rose-Bengal test as a screening test is fair compared to more accurate serological tests such as ELISA; and the disease constitutes a public health concern in the city. Additional studies are important to identify the overlooked predisposing factors, estimate the abortion rate attributable to brucellosis in food-producing animals, and evaluate efficacy of vaccination programs in reducing the prevalence of brucellosis and/or abortion rate.

## Introduction

Brucellosis is an endemic disease in food-producing animals in Mosul, Iraq. In a very first preliminary study used 3,255 cattle, 1,060 sheep, 845 goats and 404 combined sheep and goat from different areas in northern of Iraq, including Nineveh governorate, the positive animals constituted 3.1%, 1%, 4.4%, and 2%, respectively [1]. In a subsequent study, 0.78% of examined sheep and 2.55% of goats in Mosul tested seropositive using Brewer’s card test [2]. Later, number of animals tested seropositive in the city, however, has been increased. For instance, Hadad and Al-Azawy [3] identified 5.5% sheep and 5.3% goats seropositive for *Brucella* spp. antibodies using Rose Bengal Test (RBT) representing 91 flocks from Nineveh. On the other hand, Al-Farwachi et al. [4] documented 16.7% of cattle tested seropositive using c-ELISA. In addition, Al-Iraqi et al. [5] reported that 50% of buffaloes that were tested using c-ELISA were seropositive.

Brucellosis constitutes burden to animal producers in Mosul, Nineveh governorate, Iraq. In a most recent available official statistics, the total number of food-producing animals in Nineveh was 1,466,078 including 1,247,225 sheep, 114,000 goats, 78,668 cattle, 13,961 buffaloes, and 12,224 camels [6]. In that statistics, Nineveh was the second governorate in the list in the population of food-producing animals, after including Kurdistan region. Abortion rates in ewes and buffalo flocks affected with brucellosis in Mosul were estimated at 17.6% and 6.7%, respectively [7]. In a more recent study, the abortion rate in two flocks of sheep was 11.7% [8]. Moreover, in two studies conducted on aborted ewes used RBT as a detection method, one study found 18.5% of aborted ewes tested seropositive for brucellosis [9], and the other study reported that 70% of aborted ewes tested seropositive for brucellosis [10]. Control programs, however, have been poorly implemented. Vaccination program that included vaccination of lambs at 3–6 month-old and calves at 6–8 month-old has been started in 2007 by Veterinary Directorate. Nevertheless, it has been interrupted between 2014 and 2017 due to the military situation in the region. Though, efficacy of that program has not been studied.

Brucellosis is considered a public health concern issue in Mosul, Iraq. Different studies isolated *Brucella* spp. from animal-products used for human consumption in Mosul. In one study, 11 isolates (7%) of *Brucella* spp. were isolated from sheep-milk products [11]. In another study, 68% of sheep-milk samples contained DNA for *Brucella melitensis* identified using polymerase chain reaction (PCR) technique [10]. In human, Mosul records significant infection rate of brucellosis [12]. In a recent seroprevalence that included 385 patients showed symptoms of brucellosis in the city, 29.9% was positive, where female patients showed higher prevalence compared to males [13]. In that study, the prevalence of brucellosis in patients with ages 31–40 year-old was greater than that reported for older or younger ages. Potential source of infection, in addition to handing infected animals, is consumption of traditional homemade cheese that is usually not pasteurized, and “Kishfa” or “Gushwa”; the upper layer of boiled sheep milk, which is considered a good medium for incubation of *Brucella* spp. [12].

A narrative review for studies that have serologically investigated brucellosis in farm animals and human in Northern governorates in Iraq between 1974 and 2004 was conducted [14]. That review concluded that the infection rates of brucellosis in both animals and humans have dramatically increased, without existence of successful program for control and eradication [14]. Although different studies have been performed in Mosul, Iraq to report the prevalence of brucellosis, use of different serological or molecular tests for the diagnosis, or isolate *Brucella* spp. from suspected animals or specimens, a systematic review or meta-analysis that can assess and summarize those studies has not been conducted. The study conducted here was performed to achieve two objectives: (i) to identify and assess the evidence and knowledge gaps in published studies that have examined brucellosis in different food-producing animals in Mosul, Iraq; using systematic review approach, and (ii) to quantify the seroprevalence of brucellosis in food-producing animals in Mosul, Iraq; using meta-analysis approach.

## Methods

A systematic review and meta-analysis for studies that have examined brucellosis in different food-producing animals (including sheep, goats, cattle, and buffaloes) in Mosul, Iraq was conducted according to Preferred Reporting Items for Systematic reviews and Meta-Analyses (PRISMA) statement [15]. In this study, brucellosis was defined as an infection with *Brucella* spp. detected in food-producing animals using serological or molecular methods, or isolation of *Brucella* spp. from animal specimens or animal products.

### Eligibility criteria

Studies written in Arabic or English, published in peer-reviewed journals, presented original data collected from animals of interest in Mosul (and different areas around Mosul inside Nineveh governorate), Iraq, were considered for inclusion. Master theses and PhD dissertations were also considered for inclusion in this study. Narrative reviews or meta-analysis were excluded.

In this systematic review, PICOS approach was used to identify characteristics of qualified studies [16], including: (i) P: population: food-producing animals including sheep, goats, cattle, and buffaloes in Mosul (and different areas around Mosul inside Nineveh governorate), Iraq; (ii) I: intervention (or exposure): brucellosis, provided that brucellosis is an infection with *Brucella* spp. detected using serological or molecular methods, or isolation of *Brucella* spp. from animal specimens or animal products, or vaccination in clinical trials, (iii) C: comparators: those animals tested negative for brucellosis, or groups (i.e., subcutaneous vaccines vs. conjunctival drops) in clinical trials; (iv) O: outcome: result of tests used for the diagnosis, *Brucella* spp. isolates, or antibody titers; and (v) S: study design: observational studies that reported prevalence of brucellosis, used different serological or molecular tests for the diagnosis, or isolated *Brucella* spp. from suspected animals or specimens, or clinical trials that assessed vaccines against brucellosis.

### Information sources and selection process

Google Scholar was used as a search engine to track pertinent peer-reviewed research reports. In this type of search, Google Scholar can track more pertinent reports than other search engines as it is a regulation in the University of Mosul that the faculty members upload their research reports to their Google Scholar accounts.

**Fig 1:**
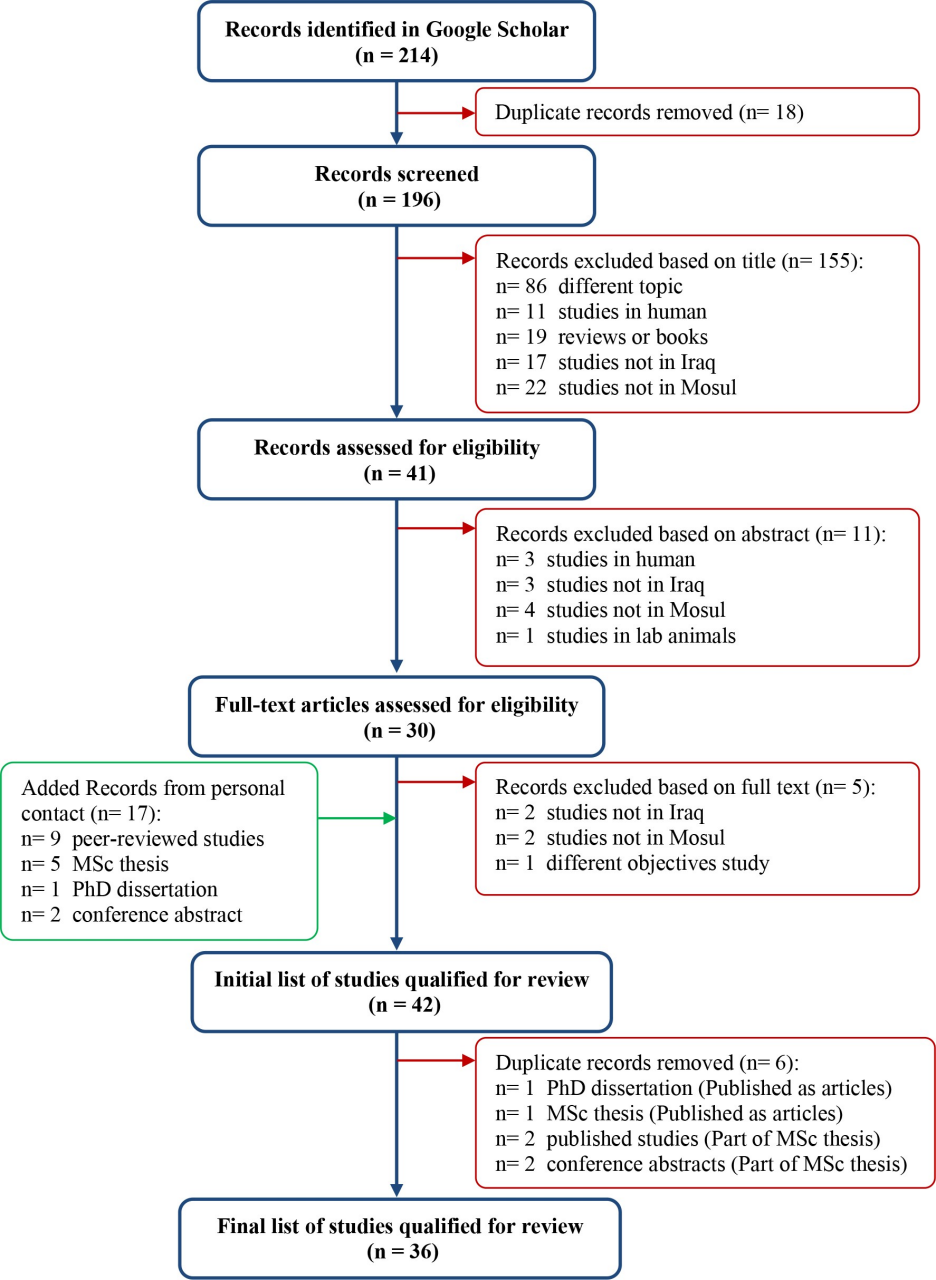
Flow diagram of articles selection process applied in the systematic review.

The search was conducted on November 24, 2019. Keywords used were: brucella, animal, Mosul, Iraq. Selected keywords were entered in the search box as a phrase where each word was followed by comma and one space as the following: (brucella, animal, Mosul, Iraq). Duplicate records were removed, and the screening process was conducted at three levels: titles, abstracts, and full-text articles in order to determine the final number of articles qualified for review (Fig 1). Additional pertinent peer-reviewed research reports obtained from personal contact or qualified articles’ references list were added (Fig 1).

### Data collection

The following data were collected: study objective/s, animal species, study sample size, number of animals tested positive for brucellosis, test/s used for the diagnosis, agreement between screening tests used for the diagnosis, number of isolates, *Brucella* spp. that were isolated, type of vaccine used, administration method of the vaccine, and the main conclusion of the study that assessed the vaccines.

### Assessment of qualified studies

Qualified studies were divided into four main groups on a basis of the study objective: (i) studies that reported prevalence of brucellosis; (ii) studies that assessed different screening tests for diagnosis of brucellosis, taking into account excluding confirmative tests such as tube agglutination test (TAT) and 2-mercapto-ethanol tests (2ME) because they were usually performed on samples tested positive in screening tests; (iii) studies that isolated *Brucella* spp. from animal specimens or animal products; and (vi) studies that assessed vaccines against brucellosis.

Qualified studies were assessed according to methods used in each study type as the following: (i) studies that reported prevalence of brucellosis were assessed based on principles of methodology for prevalence studies [17, 18] including sample size, sampling methods, standardized methods used for the diagnosis, and risk factors examination; (ii) studies that assessed tests used in the diagnosis were evaluated based on using same sample in both tests, control of workup bias between tests results (i.e., use of masking during tests implementation), and methods used for measurement of the agreement; (iii) studies that isolated *Brucella* spp. were assessed on a basis of aseptic sampling technique, using an appropriate media for culture, and specific identification of *Brucella* spp.; and finally (iv) studies that assessed vaccines against brucellosis were considered as clinical trials, and thus assessed based on sample size calculation, enrollment of the animals in the trials, bias control (including randomization and masking), and baseline data comparison [19].

### Meta-analysis

In this study, research reports obtained from the systematic review conducted here reported the seroprevalence of brucellosis were included in a meta-analysis in order to calculate an overall prevalence. Studies that targeted specific populations (i.e., aborted ewes only, rams only, young animals only) were excluded from the analysis because they negate the assumption of Cochran’s Q statistics of homogeneity that subjects are selected at random from a larger population. In this study, seroprevalence of brucellosis represented the number of animals that tested seropositive for brucellosis in identified studies over the total number of animals that were tested. When a study used more than one screening test for the diagnosis (e.g. RBT and enzyme-linked immunosorbent assay (ELISA) technique), the more accurate test estimation, e.g., ELISA [5, 20], was considered in the meta-analysis. A total of 25 studies published between 1979 and 2019 was used in the analysis. Extracted data included type of animals used, study sample size, and number of animals tested positive for brucellosis.

Serum samples testing results for a total of 18,103 animals (11,549 sheep; 3,219 goats; 2,563 cattle; and 772 buffalo) were used to estimate the overall seroprevalence. A random-effects model was used based on the assumption that the variation of the seroprevalence across studies is true [21]. Cochran’s Q statistic was used to assess the evidence of heterogeneity, and I-square statistic was used to describe the percentage of the variability due to existence of heterogeneity rather chance [22]. Egger regression was used to evaluate the bias [23]. The overall seroprevalence (%), 95% CI, and *P*-value were reported. To calculate the seroprevalence in each animal species (i.e., sheep, goats, cattle, and buffaloes) and decade (i.e., 1979–1989, 1990–1999, 2000–2009, and 2010–2019), meta-analysis was stratified according to the variables of “animal species” and “decade”, respectively. In the analysis, *P*-value of ≤ 0.05 was considered significant. Finally, the analysis was performed using STATA version 13.0 (StataCrop., College Station, TX, USA).

## Results

### Systematic review

A total of 214 records were initially identified using Google Scholar. Seventeen research reports were added from personal contact and qualified articles’ references list. Thirty six articles were qualified for review after removing 35 duplicate records, 155 titles, 11 abstracts, and 5 full text articles (Fig 1). The selected articles included 17 studies that reported the prevalence of brucellosis (Table 1). Eleven studies assessed different serological tests for diagnosis of brucellosis including Rose-Bengal plate test (RBT), modified Rose-Bengal plate test (mRBT), indirect enzyme-linked immunosorbent assay (i-ELISA), competitive enzyme-linked immunosorbent assay (c-ELISA), as well as polymerase chain reaction (PCR) (Table 2). In addition, Nine studies isolated *Brucella* spp. (mainly *B*. *abortus* and *B*. *melitensis*) from animal specimens and/or animal products including blood, milk, vaginal swabs, aborted fetuses, kishfa (the upper layer of boiled sheep milk), and cheese made in traditional method (Table 3). Finally, four studies assessed vaccines against brucellosis using *B*. *melitensis* strain Rev.1 administered subcutaneously or as conjunctival drops in ewes or goats (Table 4).

**Table 1:** Qualified studies that examined brucellosis in different food-producing animals in Mosul, Iraq, with main objective: Reporting the prevalence of the disease.

Study	Animal	Sample	Positive	Tests used
Karim et al., [2]	1. Sheep	Serum; (n = 1,037)	8 (0.77%)	BCAT
	2. Goats	Serum; (n = 1,179)	30 (2.5%)	BCAT
Al-Dahash et al., [24]	Sheep	Serum; (n = 1,621)	80 (4.9%)	RBT
Hadad and Al-Azawy, [3]	1. Sheep	Serum; (n = 2,161)	119 (5.5%)	RBT
	2. Goats	Serum; (n = 1,008)	53 (5.3%)	RBT
Hadad and Jamalludeen, [25]	Cattle	Serum; (n = 2006)	117 (5.8%)	RBT
Al-Khafaji and Rhaymah, [26]	Sheep	Not mentioned; (n = 225,534) ^1^	(2.17%)	Not mentioned
Hussain et al., [7]	1. Buffalo	Serum; (n = 240)	15 (6.3%)	RBT
	2. Cows	Serum; (n = 112)	12 (10.7%)	RBT
	3. Ewes	Serum; (n = 184)	24 (13%)	RBT
Mansour, [27] ^2^	1. Sheep	Serum; (n = 1,918)	136 (7.1%)	RBT
	2. Goats	Serum; (n = 385)	41 (10.6%)	RBT
	4. Cattle	Serum; (n = 193)	13 (6.7%)	RBT
Saleem et al., [28]	Sheep	Serum; (n = 2038)	271 (13.3%)	RBT
Hassan et al., [9]	Aborted ewes	Serum; (n = 400)	74 (18.5%)	RBT
Al-Aalim et al., [29]	Goats ^3^	Serum; (n = 184)	56 (30.4%)	RBT
Al-Obaidi et al., [30]	1. Sheep	a. Serum; (n = 211)	18 (8.5%)	RBT
		b. Milk; (n = 211)	14 (6.6%)	i-ELISA
	2. Goats	a. Serum; (n = 88)	6 (6.8%)	RBT
		b. Milk; (n = 88)	10 (11.3%)	i-ELISA
Al-Hussary and Al-Zuhairy, [31]^4^	Sheep	Serum; (n = 96)	23 23.96%)	RBT
Al-Abdaly et al., [32]	Buffalo	Serum; (n = 400)	52 (13%)	RBT
Isihak et al., [33]	Sheep ^5^	Serum; (294)	111 (37.8%)	RBT
Al-Dabagh et al., [34]	Aborted ewes	Serum; (100)	56 (56%)	ELISA
Alsanjary et al., [10] ^6^	Aborted ewes	1. Serum; (50)	35 (70%)	RBT
		2. Milk; (50)	34 (68%)	PCR
Al-Hankawei et al., [35]	Rams	Serum; (250)	12 (4.8%)	i-ELISA

Abbreviations: (BCAT): Brewer's card agglutination test, (RBT): Rose Bengal plate test, (i-ELISA): Indirect enzyme-linked immunosorbent assay.

1 This study used records of seven veterinary hospitals for 10 years (between 1980 and 1990).

2 MSc thesis, part of it published as an abstract in a conference [36].

3 Included aborted does (n = 17), vaccinated does (n = 25), and unvaccinated does (n = 142).

4 The main objective of this study was evaluating biochemical parameters in sheep infected with toxoplasmosis and brucellosis.

5 Included aborted ewes (n = 50), vaccinated ewes (n = 55), and unvaccinated ewes (n = 189).

6 The main objective of this study was to detect *B*. *melitensis* in aborted ewes’ milk using PCR.

**Table 2:** Qualified studies that examined brucellosis in different food-producing animals in Mosul, Iraq, with main objective: Assessing different screening tests for the diagnosis.

Study	Animal	Sample	Tests used	Positive	Agreement
Al-Hankawe, [37] ^1^	1. Sheep	Serum; (n = 364)	RBT	43 (11.8%)	74.1% w/ i-ELISA
			mRBT	49 (13.5%)	84.5% w/ i-ELISA
			i-ELISA	58 (15.9%)	
	2. Goats	Serum; (n = 273)	RBT	24 (8.8%)	48% w/ i-ELISA
			mRBT	83 (13.9%)	76% w/ i-ELISA
			i-ELISA	50 (18.3%)	
Mohammed, [38] ^2^	Sheep	Serum; (n = 485)	RBT	185 (38.1%)	*
		Serum; (n = 30)	PCR:		
			OMP2	28 (93.3%)	91.7% w/+ve RBT
			B4/B5	30 (100%)	100% w/+ve RBT
			*B*. *melitensis*	26 (86.7%)	91.7% w/+ve RBT
Al-Khafaji, [39] ^3^	Sheep	Serum; (n = 912)	RBT	101 (11.1%)	80% w/ c-ELISA.
			c-ELISA	50 (5.5%)	
Al-Farwachi et al., [4]	Cattle	Serum; (n = 126)	c-ELISA	21 (16.7%)	Kappa = 0.229
			RBT	23 (18.3%)	
Al-Obaidi et al., [30]	1. Sheep	a. Serum; (n = 211)	RBT	18 (8.5%)	77.6%
		b. Milk; (n = 211)	i-ELISA	14 (6.6%)	
	2. Goats	a. Serum; (n = 88)	RBT	6 (6.8%)	60.1%
		b. Milk; (n = 88)	i-ELISA	10 (11.3%)	
Al-Iraqi et al., [5]	Buffalo	Serum; (n = 132)	c-ELISA	67 (50.8%)	Kappa = 0.353
			RBT	38 (28.8%)	
Arslan et al., [20]	Sheep	Serum; (n = 228)	RBT	20 (8.7%)	Kappa = 0.38
			i-ELISA	54 (23.6%)	
Arslan et al., [40]	Goats ^4^	Serum; (n = 102)	RBT	7 (6.8%)	Kappa = 0.30
			i-ELISA	25 (24.5%)	
Mohammed et al., [41]	1. Lambs	Serum; (n = 95)	RBT	0 (0%)	0%
			i-ELISA	24 (25.3%)	
	2. Kids	Serum; (n = 40)	RBT	0 (0%)	0%
			i-ELISA	11 (27.5%)	
Rhaymah et al., [42]	Cattle	Serum; (n = 126)	RBT	23 (18.25%)	**
			i-ELISA	29 (23.01%)	
Alsanjary et al., [10]	Aborted ewes	1. Serum; (50)	RBT	35 (70%)	1. Kappa = 0.206
		2. Milk; (50)	PCR	34 (68%)	2. McNemar's = 66%

Abbreviations: (RBT): Rose Bengal plate test, (mRBT): modified rose Bengal plate test, (i-ELISA): Indirect enzyme-linked immunosorbent assay, (PCR): Polymerase chain reaction, (c-ELISA): Competitive enzyme-linked immunosorbent assay.

1 MSc thesis, part of it published as an article in a conference [43].

2 MSc thesis, published as an abstract in a conference [44].

3 MSc thesis, part of it published as an article [45].

4 Same animals were also used in another study [46] aimed to study the biochemical changes in goats affected with brucellosis.

* The agreement was 94.4% with–ve RBT using OMP2 in PCR, 100% with–ve RBT using B4/B5 in PCR, and 83.3% with–ve RBT using *B*. *melitensis* in PCR.

** Agreement of i-ELISA with RBT was 79.31% with positive RBT and 94.17% with negative RBT samples.

**Table 3:** Qualified studies that examined brucellosis in different food-producing animals in Mosul, Iraq, with main objective: Isolation of *Brucella* spp.

Study	Animal	Sample Type	Culture positive	*Brucella* spp. isolated by culture
Hadad and Al-Azawy, [47]	Sheep	Milk; (n = 3)	1	All were *B*. *melitensis*
		Aborted fetus; (n = 21)	7	
		Vaginal swab^1^; (n = 6)	4	
		Synovial fluid^2^; (n = 1)	1	
Hadad and Jamalludeen, [48]	Cattle^3^	Milk; (n = 19)	3	18 isolates *B*. *abortus*, and 3 isolates *B*. *melitensis*
		Aborted fetus; (n = 10)	4	
		Vaginal swab; (n = 6)	13	
Hadad et al., [11]	Products	Kishfa^4^ (n = 65)	3	*B*. *melitensis*
		Cheese^5^ (n = 85)	8	*B*. *abortus*
Hassan et al., [9]	Aborted ewes	Vaginal swab^6^	54	Not mentioned
Al- Hankawe, [37]	Sheep and goat	1. Aborted fetus; (n = 14)	7	1 isolate *B*. *abortus*, and 6 isolates *B*. *melitensis*
		2. Milk^1^; (n = 5)	0	
		3. Vaginal swab^1^; (n = 7)	0	
Mohammed, [38]	Sheep	1. Aborted fetus (n = 12)	7	1 isolate *B*. *abortus*, and 6 isolates *B*. *melitensis*
		2. Vaginal swab	0	
Al-Farwachi et al., [8]	Sheep^7^	Fetal stomach contents; (n = 12)	5	Modified ELISA, and species not mentioned
			4	
Al-Abdaly et al., [49]	Sheep	1. Milk; (30)	1	Species not mentioned
	Cow	2. Blood; (50)	0	Species not mentioned
	Buffalo	3. Vaginal swab; (n = 45)	8	Species not mentioned
		4. Fetal stomach; (n = 25)	12	Species not mentioned
		5. Fetal membrane; (n = 5)	1	Species not mentioned
		6. Fresh cheese; (n = 73)	3	
		Milk; (10)	0	
		Milk; (10)	0	

1 From recently aborted ewes.

2 Knee joint synovial fluid from a ram affected with severe arthritis.

3 Recently aborted cows tested positive in RBT.

4 Kishfa is the upper layer of boiled sheep milk.

5 Fresh soft cheese made in a traditional way.

6 From aborted ewes tested positive in RBT = 74.

7 Serum from 12 aborted ewes showed 8 (66.7%) positive in RBT, and 10 (83.3%) in iELISA.

**Table 4:** Qualified studies that examined brucellosis in different food-producing animals in Mosul, Iraq, with main objective: Assessing vaccines against brucellosis.

Study	Animal	Vaccine	Administration	Conclusion
Al-Khafaji, [39]	Ewes (n = 28)	*B*. *melitensis* strain Rev.1	1. S/C injection	S/C inject better than conjunctival drops
			2. conjunctival drops	
Al-Hankawe, [50]	Ewes (n = 20)	*B*. *melitensis* strain Rev.1	1. S/C injection	S/C inject better than conjunctival drops
			2. conjunctival drops	
Al-Khafaji, [51]	Ewes (n = 18)	*B*. *melitensis* strain Rev.1	1. S/C injection	Milk ELISA cannot differentiate between infected and vaccinated animals.
			2. conjunctival drops	
Aldabagh et al., [52]	Goats “bucks” (n = 30)	*B*. *melitensis* strain Rev.1	1. S/C injection	S/C inject better than conjunctival drops
			2. conjunctival drops	

### Meta-analysis

**Fig 2:**
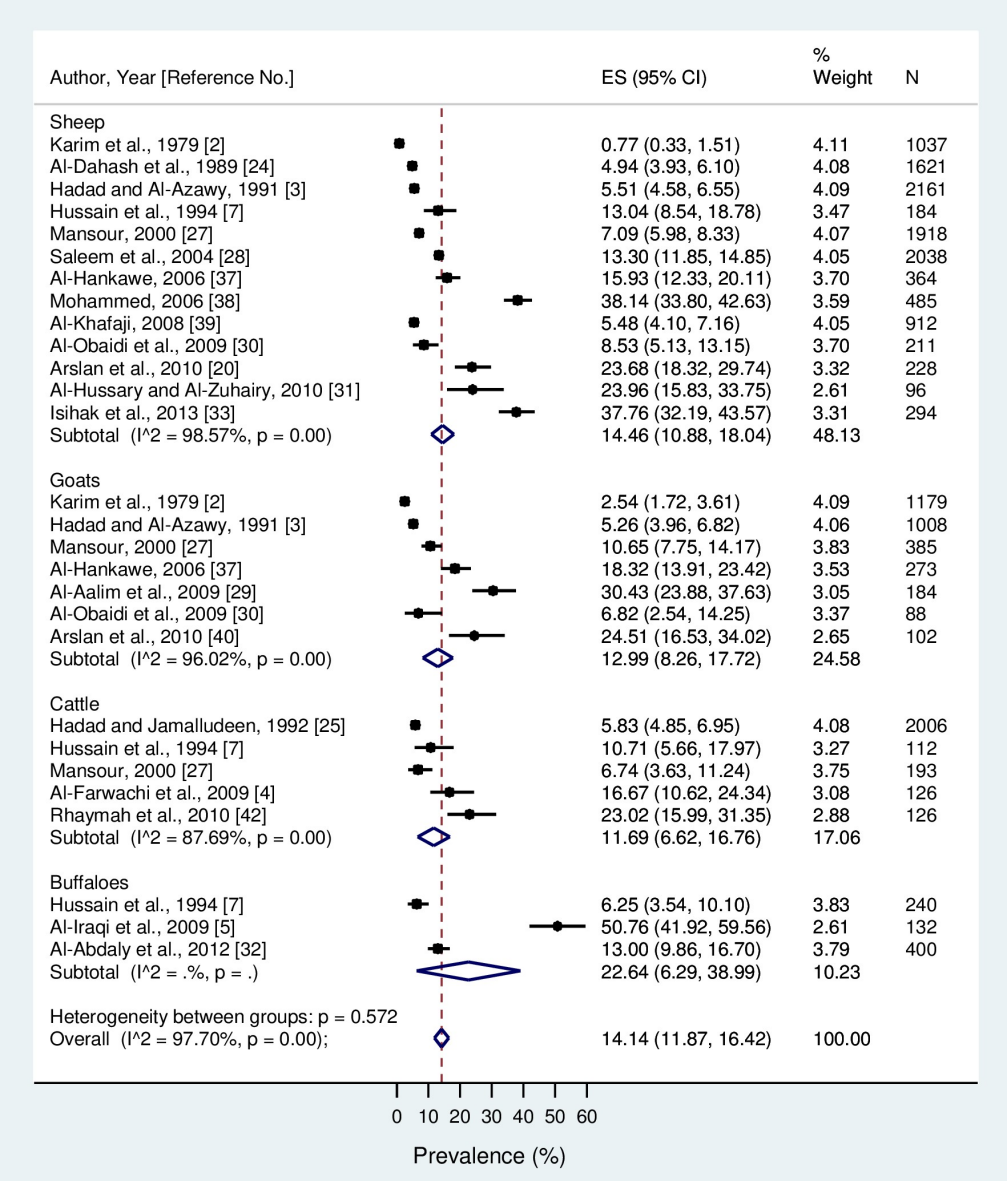
Forest plot for seroprevalence of brucellosis in food-producing animals in Mosul, Iraq.

**Fig 3:**
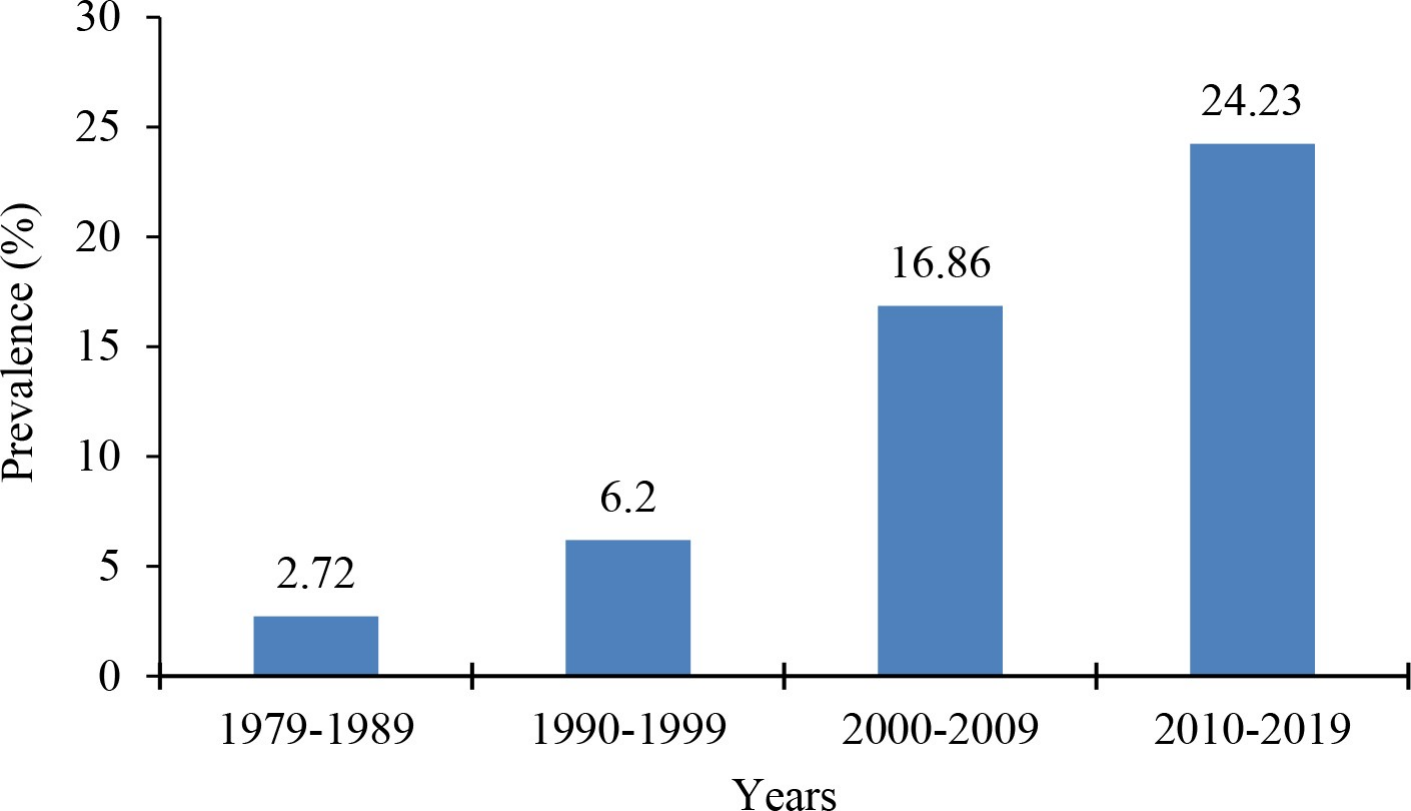
Seroprevalence of brucellosis in food-producing animals in Mosul, Iraq over a period of forty years (1979–2019).

The overall seroprevalence of brucellosis in food-producing animals in Mosul, Iraq over a period of 40 years was 14.14% (95% CI = 11.87, 16.42), including 14.46% (95%CI = 10.88, 18.04) for sheep, 12.99% (95% CI = 8.26, 17.72) for goats, 11.69% (95% CI = 6.62, 16.76) for cattle, and 22.64% (95% CI = 6.29, 38.99) for buffalo (Fig 2). The seroprevalence was increased over the four decades included in the analysis (Fig 3). The analysis showed evidence of heterogeneity (Q statistics *P*-value < 0.01). The percentage of total variation between studies due to existence of heterogeneity was high (*I*^*2*^ = 97.7%). Egger regression indicated that there is evidence of small-study effect (bias coefficient = 0.096 with a standard error of 0.035, and a *P*-value of 0.01).

## Discussion

Brucellosis has been reported in Mosul, Iraq since the 1970s of the last century. The systematic review conducted here is considered the first study that identifies and assesses the evidence and knowledge gaps in published studies examined brucellosis in food-producing animals in the city. The meta-analysis performed here is the first analysis that quantifies the seroprevalence of the disease over a period of 40 years. The study revealed a total of 36 studies examined brucellosis in sheep, goats, cattle, and buffalo in Mosul (including different areas around Mosul inside Nineveh governorate), Iraq, with an overall seroprevalence of 14.14%.

### Systematic review

#### Studies reported the prevalence of brucellosis

The systematic review identified 17 studies conducted between 1979 and 2019 that reported the prevalence of brucellosis in different animals including sheep, goats, cattle, and/or buffalo. Animals used in the identified studies included those from slaughterhouse (e.g., [2, 24, 27]), flocks (e.g., [3, 7, 28]), random animals (e.g., [27, 32]), aborted ewes (e.g., [8, 10, 34], ewes regardless of the abortion or vaccination status (e.g., [29, 30], or rams only [35]. The identified studies have focused on the standardized techniques used for collecting and handling blood samples, as well as the diagnostic tests used for classifying infected and uninfected animals. Critical appraisal to identified studies, however, reveals that principles of sampling methodology for prevalence studies have been overlooked [17, 18]. That is, all identified studies have missed the sample size calculation in their methods. Moreover, although most studies stated that they have randomly collected study animals, no study indicated the approach they used in collecting the animals randomly, particularly some studies used aborted ewes [34] or rams with signs of orchitis [35]. Therefore, the reported prevalence could have been over or underestimated.

Potential risk factors associated with brucellosis have been poorly studied in the identified research reports. Sex and age, for instance, have been examined by some studies. In one study, 6.4% ewes and 2.5% rams were positive [24]. In contrast, 10.6% ewes and 65.6% rams were tested positive in Saleem et al. study [28]. On the other hand, the highest prevalence of brucellosis was reported in sheep with age of around 3 years-old [38, 42]. Other factors, however, such as: animal species, parity, season, geographical area were not examined in the identified studies. It has been indicated that the risk of brucellosis is increased with the age [53, 54]. In addition, the risk of infection is higher in female than male, in sheep and goats than cattle and buffalo, in large size herds/flocks than small ones, and in winter than other seasons [53].

#### Studies assessed different serological test for diagnosis of brucellosis

A total of 11 studies assessed different serological tests for diagnosis of brucellosis, mainly ELISA in comparison to the traditional rapid test (i.e., RBT). The outcome in the identified studies was binary; positive/negative for brucellosis. Cohen’s kappa statistic (κ), therefore, is usually conducted to measure the agreement in this type of evaluation [55, 56]. However, only 4 studies used κ to evaluate the diagnosis using ELISA in comparison to RBT [4, 5, 20, 40]. In those studies, the estimated values of κ were between 0.229 and 0.38; indicating that the agreement was fair [55, 56]. One additional study used κ also [10]; however, the samples were different for each test; RBT and PCR used for serum and milk samples, respectively. On the other hand, the rest of the studies (i.e., [30, 37–39, 42]) have estimated the agreement between used tests by calculating the proportions of positive/negative samples in one test among positive/negative samples of the other test. Reporting both ways, i.e., κ and the agreement for positive and negative results, could provide more informative fact about the agreement between two tests than using only one method of agreement evaluation; as κ estimates the degree of the agreement [55], and the positive/negative results agreement can have clinical value, particularly when one test produces false positive/negative results [56].

#### Studies isolated *Brucella* spp. from animal specimens and/or animal products

Different *Brucella* spp. have been isolated in 9 studies conducted in Mosul, Iraq. *Brucella melitensis* was mostly isolated from sheep specimens or products including vaginal swabs, aborted fetuses, synovial fluid, milk [37, 38, 47] and kishfa [11], as well as from some cattle specimens [48]. On the other hand, *B*. *abortus* was primarily isolated from cattle specimens [48], and rarely from sheep and goats [37, 38]. These findings are in line with what is known that *Brucella* spp. have host preferences; *B*. *melitensis* infects mostly sheep and goats, and occasionally cattle, in contrast to that for *B*. *abortus* [57]. Isolation of *Brucella* spp. from vagina of aborted animals and aborted fetuses indicates the importance of the bacteria as a cause of abortion in food-producing animals in Mosul, Iraq. Furthermore, isolation of *Brucella* spp. from milk and dairy products that are usually produced locally constitutes a public health concern, particularly *B*. *melitensis*, the most commonly isolated species in the identified studies, which has been indicated as a major pathogenic species for human [58].

#### Studies assessed vaccines against brucellosis

Only 4 clinical trials have been conducted to assess vaccination procedures against brucellosis in Mosul, Iraq [39, 50–52]. All trials have examined *B*. *melitensis* Rev. 1 as a subcutaneous injection or conjunctival drops in ewes or does. No study, however, has been conducted on cattle or buffalo although two types of vaccines are usually used in those species including *B*. *abortus* S19 and RB51 [59]. Critical evaluation to those identified studies indicates that some principles of clinical trials have been overlooked, including consideration of sample size in the study design, approach used for enrollment of the animals in the trials, and bias control methods including randomization and masking [19]. Although the studies indicated that subcutaneous injection can give better antibody titers than conjunctival drops, the efficacy of the vaccination in decrease of the abortion rate has not been evaluated.

### Seroprevalence of brucellosis

The meta-analysis conducted here indicated that the overall seroprevalence of brucellosis was 14.14%. Although different vaccines have been developed and commonly used in food-producing animals [60, 61], vaccination or control programs have been poorly implemented in the region. This analysis showed that the seroprevalence in Mosul, Iraq has increased about 9 times between 1979 and 2019. The analysis, however, revealed that the heterogeneity existed with high percentage. The prevalence in identified studies was high with small study size, indicating that the heterogeneity is true [23]. The existence of heterogeneity was supported by egger regression which revealed the effect of small-studies in the analysis. The majority of recent studies were small-studies targeted specific populations with potential high burden of brucellosis, particularly in sheep, such as rams affected with orchitis [35] and aborted ewes [10, 31, 34]. Thus, the increase in the seroprevalence could have been not true. Nevertheless, different reasons can support the true increase in the prevalence of brucellosis in the city over the years including: increase the number of animals with lack of active control programs against brucellosis, free movement of animals between different areas inside and around the city, and poor herd management such as unhygienic discard for aborted fetuses. Finally, buffalo showed higher seroprevalence than that for sheep, goats, and cattle. In Pakistan, Nasir et al. [62] reported higher seroprevalence of brucellosis in buffalo than that for cattle in both government and private farms. However, the reason of high seroprevalence of brucellosis in buffalo has not well been explained in the literature, suggesting the need for more studies.

## Conclusions

### Overall current evidence

The study conducted here revealed important information related to brucellosis in food-producing animals in Mosul, Iraq including: (i) the disease is evident in the city with increasing trends over the years, although the reported prevalence could have been over or underestimated as majority of identified studies have overlooked some epidemiological tools in their methodology; (ii) buffalo shows high seroprevalence; (iii) the degree of agreement of RBT as a screening test is fair compared to more accurate serological tests such as ELISA; and (iv) brucellosis constitutes a public health concern in the city as *Brucella* spp. have been isolated from milk and dairy products.

### Knowledge gaps

Although different studies examined brucellosis in Mosul, Iraq, several knowledge gaps have been identified including: (i) complete epidemiologic situation of brucellosis in the area is not well revealed, including predisposing factors such as animal species (e.g., buffalo), animal breed, parity, pregnancy stage, season, and geographic area; (ii) abortion rate attributable to brucellosis in food-producing animals is not clearly identified; (iii) efficacy of vaccination program in reducing the prevalence of brucellosis and/or abortion rate is not evaluated.

### Policy options

In brucellosis, there is not known effective antimicrobial treatment that can cure the infected food-producing animals. Therefore, attempts are applied to reduce the infection rate. In Iraq, brucellosis is considered an endemic disease. Consequently, the first step would be selection of appropriate diagnostic tests that have high specificity and negative predictive value to reduce the number of animals tested false positive; thus, unnecessary elimination of animals is avoided. Next, use of vaccination programs in different animals, particularly young animals, to reduce the infection rate, with annual evaluation programs efficacy. An additional possible option is identification of the areas with high prevalence to restrict the movement of animals out of such areas to reduce the spread of the infection. Finally, introduce of a surveillance program of brucellosis is an important step to track the disease in different animals including wide range of areas inside and around Mosul, Iraq.

## Supporting information

S1 Checklist PRISMA 2009 checklist. (DOC)
